# Supplementary material for: End Sequence Analysis Toolkit (ESAT) expands the extractable information from single-cell RNA-seq data
Source: Genome Res. 2016 Oct;26(10):1397–410. doi: 10.1101/gr.207902.116 (PMC5052061; doi:10.1101/gr.207902.116)
Supplement: Supplemental Material [file supp_gr.207902.116_Supp_Materials.tar.gz › ESAT-esat_v0.1/doc/html/classumms_1_1core_1_1model_1_1score_1_1_window_all_score-members.html]

ESAT: Member List


ESAT


- Main Page
- Related Pages
- Packages
- Classes
- Files

- Class List
- Class Index
- Class Hierarchy
- Class Members

All Classes Namespaces Files Functions Variables Enumerator Pages

- umms
- core
- model
- score
- WindowAllScore

umms.core.model.score.WindowAllScore Member List

This is the complete list of members for umms.core.model.score.WindowAllScore, including all inherited members.

|  |  |  |
| --- | --- | --- |
| AbstractWindowScore(Annotation t) | umms.core.model.score.WindowScore.AbstractWindowScore |  |
| annotation | umms.core.model.score.WindowScore.AbstractWindowScore | protected |
| asRPKM(double count, double total, int windowSize) | umms.core.model.score.CountScore | static |
| compareTo(CountScore o) | umms.core.model.score.CountScore |  |
| CountScore(Annotation a) | umms.core.model.score.CountScore |  |
| CountScore(CountScore other) | umms.core.model.score.CountScore |  |
| CountScore(CountScore other, Annotation newAnnotation, double newCount) | umms.core.model.score.CountScore |  |
| CountScore(AnnotationCollection<?extends Annotation > model, Annotation annotation) | umms.core.model.score.CountScore |  |
| CountScore(AnnotationCollection<?extends Annotation > model, Annotation annotation, double regionTotal) | umms.core.model.score.CountScore |  |
| CountScore(AnnotationCollection<?extends Annotation > model, Annotation annotation, boolean fullyContained) | umms.core.model.score.CountScore |  |
| CountScore(AnnotationCollection<?extends Annotation > model, Annotation annotation, double regionTotal, boolean fullyContained) | umms.core.model.score.CountScore |  |
| CountScore(Annotation annotation, double count, double regionTotal, double total) | umms.core.model.score.CountScore |  |
| DEFAULT\_REGION\_TOTAL | umms.core.model.score.CountScore | static |
| equals(Object o) | umms.core.model.score.CountScore |  |
| getAnnotation() | umms.core.model.score.WindowScore.AbstractWindowScore |  |
| getCount() | umms.core.model.score.CountScore |  |
| getDenominatorCount() | umms.core.model.score.WindowAllScore |  |
| getDenominatorLambda() | umms.core.model.score.WindowAllScore |  |
| getDenominatorRegionTotal() | umms.core.model.score.WindowAllScore |  |
| getDenominatorRPKM() | umms.core.model.score.WindowAllScore |  |
| getDenominatorScanPVal() | umms.core.model.score.WindowAllScore |  |
| getDenominatorTotal() | umms.core.model.score.WindowAllScore |  |
| getDepletionBinomialPValue() | umms.core.model.score.WindowAllScore |  |
| getEnrichmentBinomialPValue() | umms.core.model.score.WindowAllScore |  |
| getLog2Ratio() | umms.core.model.score.WindowAllScore |  |
| getLog2RegionRatio() | umms.core.model.score.WindowAllScore |  |
| getNumeratorCount() | umms.core.model.score.WindowAllScore |  |
| getNumeratorLambda() | umms.core.model.score.WindowAllScore |  |
| getNumeratorRegionTotal() | umms.core.model.score.WindowAllScore |  |
| getNumeratorRPKM() | umms.core.model.score.WindowAllScore |  |
| getNumeratorScanPVal() | umms.core.model.score.WindowAllScore |  |
| getNumeratorTotal() | umms.core.model.score.WindowAllScore |  |
| getPvalue() | umms.core.model.score.CountScore |  |
| getRatio() | umms.core.model.score.WindowAllScore |  |
| getRegionDenominatorRPKM() | umms.core.model.score.WindowAllScore |  |
| getRegionNumeratorRPKM() | umms.core.model.score.WindowAllScore |  |
| getRegionRatio() | umms.core.model.score.WindowAllScore |  |
| getRegionTotal() | umms.core.model.score.CountScore |  |
| getRPK() | umms.core.model.score.CountScore |  |
| getRPKM() | umms.core.model.score.CountScore |  |
| getScore() | umms.core.model.score.WindowAllScore |  |
| getSkellamPValue() | umms.core.model.score.WindowAllScore |  |
| getTotal() | umms.core.model.score.CountScore |  |
| hashCode() | umms.core.model.score.CountScore |  |
| refreshPvalue() | umms.core.model.score.CountScore |  |
| RPKM\_OFFSET | umms.core.model.score.WindowAllScore | static |
| setCount(double count) | umms.core.model.score.CountScore |  |
| setDenominatorCount(double d) | umms.core.model.score.WindowAllScore |  |
| setDenominatorRegionTotal(double d) | umms.core.model.score.WindowAllScore |  |
| setDenominatorScanPVal(double pval) | umms.core.model.score.WindowAllScore |  |
| setDenominatorTotal(double d) | umms.core.model.score.WindowAllScore |  |
| setNumeratorCount(double d) | umms.core.model.score.WindowAllScore |  |
| setNumeratorRegionTotal(double d) | umms.core.model.score.WindowAllScore |  |
| setNumeratorScanPVal(double pval) | umms.core.model.score.WindowAllScore |  |
| setNumeratorTotal(double d) | umms.core.model.score.WindowAllScore |  |
| setRegionTotal(double regionTotal) | umms.core.model.score.CountScore |  |
| setTotal(double total) | umms.core.model.score.CountScore |  |
| toString() | umms.core.model.score.WindowAllScore |  |
| WindowAllScore(Annotation a) | umms.core.model.score.WindowAllScore |  |
| WindowAllScore(AlignmentModel numerator, AlignmentModel denominator, Annotation annotation) | umms.core.model.score.WindowAllScore |  |
| WindowAllScore(AlignmentModel numerator, AlignmentModel denominator, Annotation annotation, double numeratorRegionTotal, double denominatorRegionTotal, double rapLambda, double backgroundLambda) | umms.core.model.score.WindowAllScore |  |


---

Generated on Fri Aug 15 2014 15:11:14 for ESAT by  

 1.8.7
